# Adaptation and reliability of the readiness for inter professional learning scale (RIPLS) in the Chinese health care students setting

**DOI:** 10.1186/s12909-018-1423-8

**Published:** 2018-12-18

**Authors:** Zhewei Li, Yihan Sun, Yang Zhang

**Affiliations:** Institute for International Health Professions Education and Research, China Medical University, No. 77 Puhe Road, Shenyang North New Area, Shenyang, Liaoning Province 110122 People’s Republic of China

**Keywords:** Medical education, Health care students, Interprofessional collaboration, Readiness for inter-professional learning scale

## Abstract

**Background:**

Interprofessional collaboration (IPC) primarily aims to enhance collaborative skills and to improve the awareness of teamwork and collaborative competencies of health care students. The Readiness for Interprofessional Learning Scale (RIPLS) was used to assess such skills. The aim of this study was to adapt a Chinese version of the RIPLS among Chinese health care students and to test the psychometric properties of the modified instrument.

**Methods:**

The questionnaire was translated following a two-step process, comprising forward and backward translations and a pilot test. The Chinese version was tested on a group of students from various health care professions. Cronbach’s α coefficients were calculated for each of the four factors and also for the entire questionnaire in order to evaluate the internal consistency of the Chinese version of the RIPLS.

**Results:**

Of the 295 health care students surveyed, 282 (96.5%) completed the questionnaire. Cronbach’s α coefficient for the overall scale was 0.842. Internal consistencies within each factor were good (α > 0.70) except for the factor “Roles and Responsibilities”, where α = 0.216. Confirmatory factor analysis showed that the data fit the four-factor structure.

**Conclusion:**

The Chinese version of the RIPLS was an acceptable instrument for evaluating the attitudes of the health care students in China. The factor “Roles and Responsibilities” requires further scrutiny and development, at least in the Chinese context.

## Background

As the demand for health care services increase in modern China, it becomes more essential for high quality medical care to coordinate interdisciplinary approaches based on common goals in medical care. Different professions in health care and social care work together to share knowledge and responsibility, to solve complex disease problems, and to meet the various patient requirements [1]. Interprofessional collaboration (IPC) can not only effectively alleviate the continued global shortage of health care professionals but can also better protect the safety of patients and improve complex disease outcomes [2]. Interprofessional collaboration (IPC) in health care can be described as the capability of every health care professional to effectively embrace complementary roles within a team, to work cooperatively, to share the responsibilities for problem-solving, and to make the decisions needed to formulate and carry out plans for patient care [3]. Interprofessional education (IPE), defined as education that involves two or more professions learning with, from, and about each other to improve collaboration and the quality of care, allows students from several healthcare professions learn and work together [4]. Some scholars have proposed that effective interprofessional education could smoothly promote IPC [5, 6].

Most Chinese health professions education programs were based on independent professional teaching, which leads to a lack of interprofessional collaboration between students from different professions and a lack of trans-disciplinary cooperation opportunities. Lack of interprofessional communication and teamwork were important reasons leading to medical errors in China [7]. Hence, interprofessional collaboration and education are important for health care students [8]. Numerous medical schools all over the world offer courses on interprofessional learning [9]. Related projects which aim to nurture students’ teamwork and collaboration skills have been launched in many countrie, such as Sweden, and Germany.

A previous study indicated that student attitudes towards interprofessional learning play an important role in interprofessional education [10]. It is of great significance to measure students’ attitudes toward interprofessional learning as early as possible in order to carry out interprofessional education reforms and establish an awareness of teamwork for students in medical schools. Educators should conduct targeted guidance for students based on the data analysis and provide relevant supports for interprofessional education.

The readiness for interprofessional learning scale (RIPLS) was first published by Parsell [11] in 1999 and then revised for use with undergraduate students by McFadyen et al. [12] in 2009. The RIPLS focuses on attitudes of undergraduate health care students and healthcare professionals toward interprofessional learning. The scale has been widely used in Canada, Sweden, Brazil, Japan, and the Netherlands [13–17]. However, the application of the RIPLS is still in its initial stage in China. To facilitate the use of this tool by other researchers in China, the purpose of this study was to adapt the Chinese version of the readiness for interprofessional learning scale (RIPLS) among health care students and to test the psychometric properties of the adapted instrument.

## Methods

### Study design, participants, and procedures

The cross-sectional study was performed at China Medical University in June 2017. Thirty 2nd year clinical students at China Medical University were enrolled in the pilot study to conduct cross-cultural adaptation. A total of 295 s year students, comprising medical students (*n* = 175, not including the 30 students in the pilot study), nursing students (*n* = 61), and clinical pharmacy students (*n* = 59), were recruited from China Medical University, Shenyang, China, by cluster sampling via random numbers (50% of each program were randomly chosen by clusters). All students were asked to complete the questionnaire. To assist with survey dissemination, four investigators were recruited through the Student Association of Science and Technology. As part of their training, the investigators were familiarized with the purpose of the questionnaire and its specific content. The self-reported questionnaires were completed individually by participants, and participants were informed that their responses would remain anonymous.

### Setting

Most medical students in China are admitted from high school into a five-year medical program in Chinese medical schools comprising 2 years basic science, 2 years clinical medicine, and 1 year internship training. Nursing students and clinical pharmacy students undergo a 4-year program, including 2 years of basic science, 1 year of professional courses, and 1 year of professional practice.

### Variables and instruments

The self-administered questionnaire consisted of two parts: 1) demographic information, including items such as gender, study year, and major; and 2) a 19 item self-reported scale based on the readiness for interprofessional learning scale (RIPLS) developed by Parsell and Bligh [11]. The RIPLS was further developed into a four factor model by McFadyen et al. [12]. The RIPLS was the most frequently used instrument for assessing student attitudes toward interprofessional education. The scale is divided into four factors: “teamwork and collaboration”, “negative professional identity”, “positive professional identity”, and “roles and responsibilities”. A 5-point Likert scale was used to rate all items, with opinions ranging from ‘strongly agree’ to ‘strongly disagree’. Higher scores revealed stronger positive attitudes towards interprofessional education.

### Cross-cultural adaptation

The RIPLS was adapted for use in China from the original version based on cross-cultural adaptation guidelines originally developed by Guillemin et al. [18]. The questionnaire was translated following a two-step process, involving forward and backward translations. First, two experienced medical teachers translated the English version of the RIPLS into Chinese. Then, two bilingual English-Chinese translators blind to the original English version back-translated the temporary Chinese version into English. A final version of the Chinese RIPLS was produced by a professional bilingual medical education expert who compared both back-translated English versions with the original version of the RIPLS to ensure no differences in translation.

An expert committee comprising researchers, translators, and IPC teachers held a discussion regarding the translated version of the RIPLS, and an approved version was created for field testing. The approved Chinese version of the RIPLS was pre-tested on 30 s year clinical students at China Medical University. During the pre-testing, participants were asked to complete the Chinese version of the RIPLS. Participants were interviewed regarding their comprehension of the Chinese version of the RIPLS upon completion. Minor corrections were made to improve the sentence structure of the questionnaire instructions to make it easier to understand, and the final Chinese version of the RIPLS was completed.

### Ethical approval

The study was approved by the ethics committee of China Medical University. Students who were enrolled in this study comprised only health care students and provided written informed consent. The purpose of the study was made clear to all student participants.

### Data analysis

To minimize risk of bias, random cluster sampling was used. The study sample was chosen to be representative of the population age and sex. Investigators were trained, and both investigators and participants were informed of the purpose and content of the questionnaire in order to reduce the number of unanswered forms. Missing data were replaced by the median. Cronbach’s α coefficients were calculated for the entire questionnaire and for each factor to evaluate the internal consistency of the Chinese version of the RIPLS. Internal consistency was considered acceptable when Cronbach’s α coefficient was ≥0.70 [19]. Factor analysis using principal component analysis with direct oblimin rotation was used to determine the underlying structure of the RIPLS [20]. factors were constructed based on factor loadings, with scores reversed where necessary.

Kaiser-Meyer-Olkin (KMO) test and Bartlett’s test of sphericity were used to determine whether or not confirmatory factor analysis (CFA) could be performed. Confirmatory factor analysis was performed to test the factorial structure. Goodness of fit was evaluated using χ2, root mean square error of approximation (RMSEA), comparative fit index (CFI), and adjusted goodness-of-fit index (AGFI). A RMSEA value < 0.08 and a CFI value > 0.90 indicated a good fit. For AGFI, a value > 0.85 was considered to be an adequate model fit [21]. Test-retest and inter-rater reliability were not calculated because this study was only concerned with the internal consistency of the Chinese version of the RIPLS. Data was analyzed using SPSS version 22.0 (SPSS Inc., Chicago, IL, USA) and LISREL 8.5 for Windows. A *p*-value < 0.05 was considered to be statistically significant.

## Results

### Social-demographic characteristics of health care students

Of the 295 s year health care students invited to participate in the study, 282 (95.6%) satisfactorily completed the questionnaire. Socio-demographic characteristics of the study sample are reported in Table 1.

**Table 1:** Demographic characteristics of the study sample (*n* = 282)

Demographic factor	Number	Percentage (%)
Major		
Medical students	166	58.9
Nursing students	60	21.2
Clinical pharmacy students	56	19.9
Ethnicity		
Han Chinese	265	94.1
other Chinese ethnic minorities	17	5.9
Gender		
Male	176	62.4
Female	106	37.6

### Cross-cultural adaptation

Minor discrepancies between the two translators existed during the forward translation (e.g. the word “learning” was used interchangeably with “to study”, which have similar applications in Chinese literature). The term “health care students” was translated into “health care and services students”, which is often used for students of different specializations. All 30 students in the pre-test stage found the scale easy to understand.

### Reliability of the Chinese version of the RIPLS

The internal consistency of the Chinese version of the RIPLS was overall good (α = 0.842). Cronbach’s α estimating the internal consistency of the four factors “teamwork and collaboration”, “negative professional identity”, “positive professional identity”, and “roles and responsibilities” were α = 0.963, α = 0.853, α = 0.931, and α = 0.216, respectively. Factor items and Cronbach’s α for each factor are reported in Table 2.

**Table 2:** Items in each factor and factor internal consistency

Item	
Factor 1: Teamwork and Collaboration (α = 0.963)	
1 Learning with other students will help me become a more effective member of a health care team	
2 Patients would ultimately benefit if health care students worked together to solve patient problems	
3 Shared learning with other health care students will increase my ability to understand clinical problems	
4 Learning with health care students before qualification would improve relationships after qualification	
5 Communication skills should be learned with other health care students	
6 Shared learning will help me to think positively about other professionals	
7 For small group learning to work, students need to trust and respect each other	
8 Team-working skills are essential for all health care students to learn	
9 Shared learning will help me to understand my own limitations	
Factor 2: Negative Professional Identity (α = 0.853)	
10 I do not want to waste my time learning with other health care students	
11 It is not necessary for undergraduate health care students to learn together	
12 Clinical problem solving skills can only be learned with students from my own department	
Factor 3: Positive Professional Identity (α = 0.931)	
13 Shared learning with other health care students will help me to communicate better with patients and other professionals	
14 I would welcome the opportunity to work on small-group projects with other health care students	
15 Shared learning will help to clarify the nature of patient problems	
16 Shared learning before qualification will help me become a better team worker	
Factor 4: Roles and Responsibilities (α = 0.216)	
17 The function of nurses and therapists is mainly to provide support for doctors	
18 I am not sure what my professional role will be	
19 I have to acquire much more knowledge and skills than other health care students	

### Factor structure

The results of Kaiser-Meyer-Olkin (0.901) and Bartlett’s test of sphericity (χ2 = 3733.214, *P*-value < 0.001) indicated that the samples in this study were suitable for factor analysis. Initial exploratory factor analysis revealed factors with eigenvalues greater than 1, which accounted for 59.78% of the variance. The original validation offered a four-factor solution with 19 items. A four-factor solution was chosen based on the results reported in the rotated component matrix (Table 3).

**Table 3:** Rotated component matrix

Item	Factor			
	Teamwork and collaboration	Positive professional identity	Negative professional identity	Roles and responsibilities
1	0.843	0.243	0.013	−0.104
2	0.851	0.268	0.083	−0.126
3	0.880	0.261	−0.017	−0.104
4	0.872	0.297	0.016	−0.027
5	0.876	0.241	0.015	0.003
6	0.793	0.301	0.092	0.115
7	0.846	0.273	0.036	0.044
8	0.852	0.170	0.018	0.078
9	0.769	0.193	0.048	0.006
10	−0.351	0.282	0.727	−0.095
11	−0.403	0.277	0.704	−0.128
12	−0.341	0.394	0.698	0.025
13	0.420	−0.668	0.267	−0.136
14	0.536	−0.718	0.216	−0.095
15	0.551	−0.702	0.253	−0.078
16	0.545	−0.722	0.210	−0.098
17	0.043	−0.069	0.367	0.752
18	−0.104	−0.006	0.399	−0.391
19	0.337	−0.303	0.209	0.495

### Confirmatory factor analysis

**Fig. 1:**
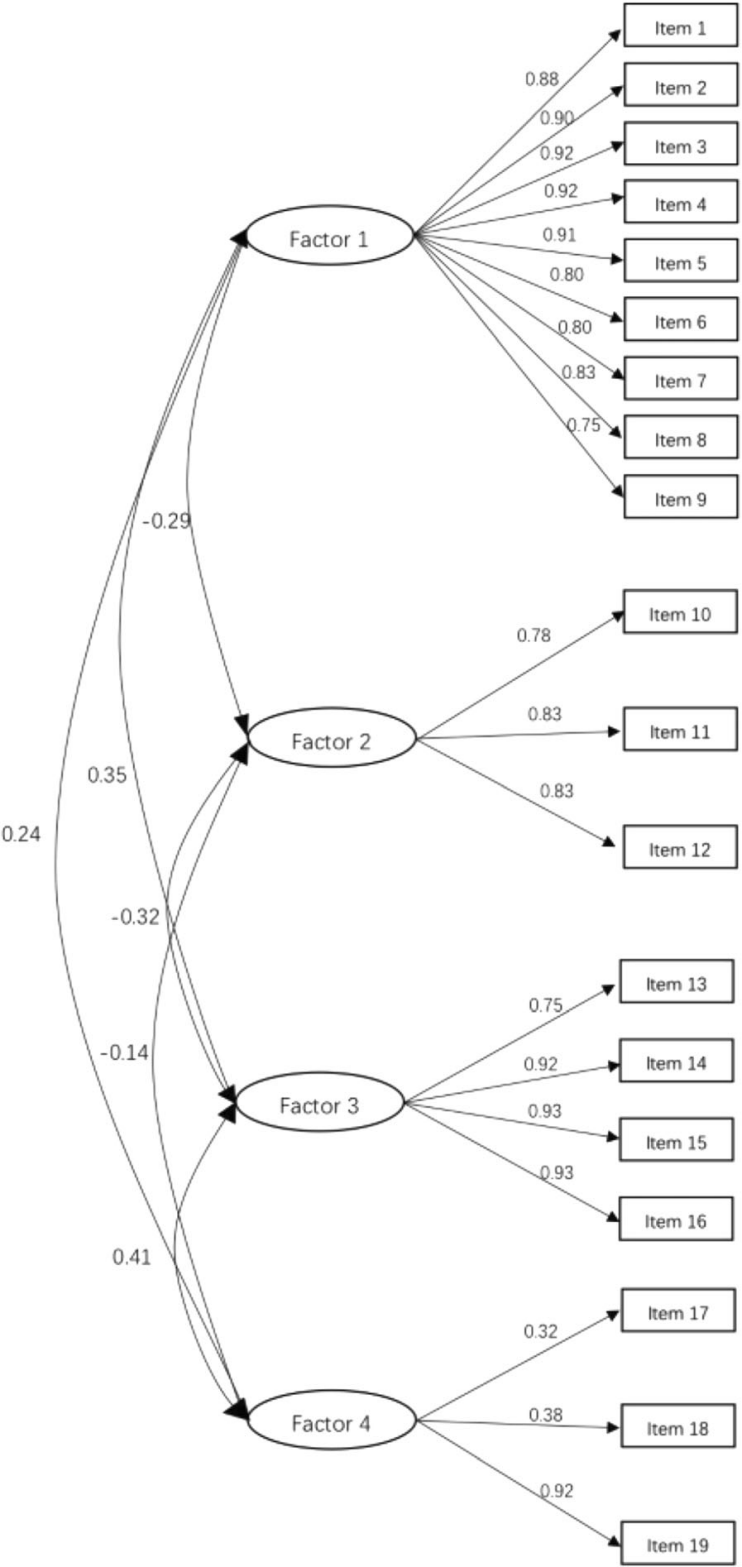
Structure of the Chinese version of the RIPLS based on confirmatory factor analysis Factor 1: Teamwork and Collaboration; Factor 2: Negative Professional Identity; Factor 3: Positive Professional Identity; Factor 4: Roles and Responsibilities

Confirmatory factor analysis showed that the four-factor structure of the RIPLS produced an acceptable fit to the data (χ2 = 2462.01, df = 220, *p* < 0.001; CFI = 0.83; RMSEA = 0.07 [90% CI: 0.04 to 0.08]; AGFI = 0.88). Factor loadings of each item with its respective domain were all acceptable, with AGFI ranging from 0.39 to 0.88 (Fig. 1).

## Discussion

This study confirmed that the adapted Chinese version of the RIPLS was a reliable and valid instrument, with acceptable psychometric properties for assessing Chinese health care students’ attitudes toward interprofessional learning. The results showed that a four-factor solution was appropriate for identifying the components of Chinese health care students’ attitudes toward interprofessional learning. The following four factors explained a major portion of the variance: “Teamwork and Collaboration”, “Negative Professional Identity”, “Positive Professional Identity”, and “Roles and Responsibilities”, of which the first three factors had good internal consistency for the items, with the exception of the factor “Roles and Responsibilities”.

While the overall RIPLS was reliable, the factor “Roles and Responsibilities” had a much lower Cronbach coefficient (α = 0.216). The results were consistent with other reliability studies in the UK (Cronbach’s α = 0.32) [11], and Germany (Cronbach’s α = 0.65) [22] for the undergraduate cohorts. The results may be attributed to fewer items within the “Roles and Responsibilities” factor, as also reported by a previous study involving graduate student cohorts [23]. McFadyen et al. reported that this was not affected by participation in interprofessional activities, and Mahler et al. postulated that professional experience was not an influencing factor due to weaker internal consistency even in graduates than in students [22]. Various potential factors could be influencing these results, so this calls for the factor “Roles and Responsibilities” to be reexamined and adjusted in future studies.

In China, clinical pharmacy is an emerging major, and the participation of professional pharmaceutical services in clinical work remains to be insufficient. The lack of awareness of and indifference [24] to professional identity in health care students and students’ idealized view of their future roles [25] may also suggest that educators should consider different perspectives on professional roles and identities when constructing and delivering interdisciplinary courses.

Another purpose of the study was to investigate whether the four-factor model from the McFadyen version of the RIPLS could be applied to the Chinese translation of the scale in a Chinese setting. The goodness-of-fit of the model was a prominent factor on the validity of the instrument. Most items showed a good goodness-of-fit, except item 18 (factor loading = 0.391). The results of the confirmatory factor analysis suggest an acceptable fit, implying that the four-factor structure of the scale can be conducted in measurements performed by the Chinese version of RIPLS.

There were a few limitations to this study. The factors of the scale proposed in the study provided helpful suggestions for promoting IPC education, but the study sample came from only one medical university in China, which may not be representative of all Chinese health care students. Further studies examining varying levels of education and participants from additional centers are needed to confirm the stability of the structure of the Chinese version of the scale.

## Conclusions

The Chinese version of the RIPLS is an overall valid and reliable instrument for evaluating the attitudes of health care students in China. However, the factor “Roles and Responsibilities” requires further analysis due to its relatively lower internal consistency.

### Ethical approval and consent to participate

The study was approved by the ethics committee of China Medical University. All participants were volunteers, freely participating without any extrinsic incentives. Only health care students who gave written informed consent were enrolled in the study.

## Abbreviations

CFA: Confirmatory factor analysis
IPC: Interprofessional collaboration
IPE: Interprofessional education
RIPLS: Readiness for Interprofessional Learning Scale
